# Associations between fear of medical imaging radiation, risk perception, and patient decisional conflict: evidence from variable-centered and person-centered analyses

**DOI:** 10.3389/fpsyt.2026.1826113

**Published:** 2026-05-13

**Authors:** Lixing Lei, Xiongxiong Yang, Xin He, Qianqian Liu, Lingling Tang, Nian Liu, Xiao-hua Huang

**Affiliations:** 1 Department of Radiology, Affiliated Hospital of North Sichuan Medical College, Nanchong, China; 2 Department of Radiology, Nanchong Hospital of Traditional Chinese Medicine, Nanchong, China

**Keywords:** decisional conflict, fear of medical imaging radiation, person-centered and variable-centered approaches, risk assessment, risk perception

## Abstract

**Background:**

Medical imaging examinations play an irreplaceable role in modern medical diagnosis; however, some patients experience decisional difficulties due to fear of radiation, potentially leading to examination delays or refusals that subsequently compromise timely disease diagnosis and treatment. The psychological mechanisms through which fear of medical imaging radiation influences patient decision-making processes remain unclear, and the heterogeneous characteristics of radiation fear manifestations across patient populations lack systematic investigation.

**Methods:**

A cross-sectional survey design was employed, with 468 adult patients scheduled to undergo medical imaging examinations conveniently sampled from two tertiary Grade-A general hospitals in Sichuan Province, China, between July and August 2025. Data were collected using the Fear of Medical Imaging Radiation Scale, Risk Perception Scale, and Decisional Conflict Scale.

**Results:**

Mediation analysis using the PROCESS macro showed that fear of medical imaging radiation was significantly and positively associated with decisional conflict. Risk perception showed a significant partial mediating role in the association between fear of radiation and decisional conflict. Latent profile analysis identified three radiation fear subtypes: a low radiation fear group, a moderate radiation fear group, and a high radiation fear group. Analysis of variance demonstrated significant differences among the three groups in both risk perception and decisional conflict, with patients in the high radiation fear group exhibiting significantly elevated levels of risk perception and decisional conflict compared with the other two groups.

**Conclusions:**

Fear of medical imaging radiation indirectly exacerbates decisional conflict by elevating patients’ risk perception levels, and significant heterogeneity exists in radiation fear manifestations across patient populations. These findings provide evidence-based guidance for healthcare institutions to develop targeted patient education programs, risk communication strategies, and psychological support interventions. The results facilitate the identification of high-risk patient populations and implementation of differentiated clinical management strategies, ultimately advancing the realization of patient-centered imaging medicine service models.

## 1 Introduction

Medical imaging technology, as a cornerstone of modern clinical diagnosis, has expanded its application scope from traditional disease screening to therapeutic efficacy evaluation, surgical planning, and prognostic monitoring (1, 2). According to World Health Organization statistics, more than 3.6 billion medical imaging examinations are performed globally each year (3, 4), with examinations involving ionizing radiation—including X-rays, computed tomography, and positron emission tomography—accounting for over 60% of this total (5). Although radiation doses from medical imaging examinations are typically maintained within safe thresholds and clinical benefits substantially outweigh potential risks, public concern regarding radiation exposure has demonstrated a sustained upward trajectory (6, 7). This concern stems partly from the imperceptibility of radiation hazards, sensationalized media coverage of nuclear radiation incidents, and patients’ limited understanding of medical knowledge (8, 9). Notably, this radiation-related fear psychology extends beyond mere emotional responses and has begun to exert substantive effects on patients’ medical decision-making behaviors. Clinical observations indicate that some patients delay or refuse necessary imaging examinations due to excessive radiation risk concerns, consequently leading to delayed disease diagnosis, missed treatment opportunities, and other adverse outcomes (10). Therefore, systematically investigating the psychological mechanisms underlying fear of medical imaging radiation and its impact on patient decision-making processes holds significant importance for enhancing healthcare service quality and physician-patient communication efficiency.

Fear of medical imaging radiation, as a manifestation of health anxiety within a specific context, encompasses three core dimensions: cognitive, affective, and behavioral (11, 12). From the cognitive perspective, patients frequently exhibit a marked tendency to overestimate radiation doses in medical imaging examinations, conflating low-dose diagnostic radiation with high-dose therapeutic radiation or nuclear accident radiation, thereby forming irrational risk cognition schemas (13). The affective dimension manifests as persistent worry about radiation exposure, anticipatory anxiety regarding examination procedures, and fearful rumination about potential health consequences (14, 15). At the behavioral level, radiation fear may drive patients to adopt avoidance behavioral strategies, such as repeatedly seeking non-radiation alternative examination options, excessively demanding physician assurances of radiation safety, or outright refusing to undergo necessary imaging examinations (10). Previous research has preliminarily revealed associations between fear of medical imaging radiation and factors including patient personality traits, prior medical experiences, health literacy levels, and social support (16, 17). However, the existing literature lacks systematic theoretical elucidation and empirical examination of the core question: how radiation fear influences patient decisional conflict through specific psychological pathways. This research gap constrains the targeted development of clinical intervention strategies and impedes the effective implementation of shared decision-making models in the medical imaging domain. Consequently, this study seeks to address two questions: (1) Is fear of medical imaging radiation positively associated with decisional conflict? (2) What are the underlying mechanisms between these two constructs?

Decisional conflict refers to a state of psychological dilemma experienced by individuals when confronting multiple viable options, arising from information uncertainty, value conflicts, or emotional ambivalence (18, 19). In healthcare contexts, the degree of decisional conflict directly relates to patient acceptance of diagnostic and treatment plans, treatment adherence, and long-term health outcomes (20, 21). When patients face decisions regarding whether to undergo imaging examinations involving radiation exposure, their decision-making process entails weighing the diagnostic value of examinations against potential radiation risks (22, 23). In this process, radiation fear may exacerbate decisional conflict through multiple pathways: on one hand, fear emotions may impair patients’ information processing capacity, rendering them unable to objectively evaluate examination necessity and safety (24); on the other hand, fear may amplify the expectation weight patients assign to negative outcomes, generating systematic bias in their risk-benefit assessments. Furthermore, cognitive resource depletion under fear states may undermine effective communication between patients and healthcare teams, further intensifying decisional uncertainty arising from information asymmetry (25). Therefore, in-depth analysis of the mechanisms linking radiation fear and decisional conflict holds substantial theoretical value and practical significance for optimizing patient informed consent processes and enhancing the quality of shared decision-making between physicians and patients.

Risk perception, as a core psychological variable in health behavior decision-making, may play a critical mediating role in the relationship between fear of radiation and decisional conflict. Prior research has demonstrated that public subjective risk assessment is not based solely on objective probability information but is profoundly influenced by risk characteristic attributes such as voluntariness, controllability, familiarity, and catastrophic potential (26, 27). Medical radiation, due to its imperceptibility, delayed effects, and potential association with cancer, is often categorized by the public as a dreaded risk—even when its actual probability of occurrence is extremely low, it may nonetheless trigger intense subjective risk perception (28, 29). Radiation fear may first activate patients’ risk cognition schemas regarding radiation hazards, subsequently amplifying perceived severity and perceived susceptibility, thereby elevating their overall risk assessment of examinations (30). This amplified risk perception may then conflict with patients’ evaluation of examination benefits, ultimately exacerbating decisional dilemmas (31). Concurrently, radiation possesses characteristics of invisibility, uncontrollability, and potential catastrophic consequences—attributes that readily trigger individuals’ instinctive fear responses, causing their risk perception to deviate from objective statistical data. Risk perception encompasses two dimensions: cognitive appraisal (rational judgment of occurrence probability) and affective response (intuitive feelings about consequences) (32, 33). When patients confront imaging examination decisions, if the anxiety of the affective dimension overwhelms the rational analysis of the cognitive dimension, patients may remain fixated on catastrophic imagery of “radiation-induced cancer” even when physicians provide comprehensive scientific explanations. Both the Health Belief Model and Protection Motivation Theory support the hypothesis of risk perception’s mediating role in the emotion-behavior chain (34, 35); however, this hypothesis has not yet received sufficient empirical support in the specific context of fear of medical imaging radiation. More importantly, existing studies have predominantly employed variable-centered analytical perspectives to examine correlational relationships between pairs of variables, neglecting the potential heterogeneity in radiation fear patterns across patient populations—a limitation that constrains the guidance value of research conclusions for developing personalized intervention strategies.

Based on the foregoing analysis, this study aims to construct and test an integrative theoretical model of how fear of medical imaging radiation influences patient decisional conflict while simultaneously exploring potential heterogeneous subtypes in radiation fear characteristics across patient populations. The study employed a cross-sectional quantitative design combining variable-centered and person-centered analytical approaches, specifically regression-based mediation analysis using the PROCESS macro and latent profile analysis, seeking to advance theoretical understanding at both the level of variable relationship verification and individual difference identification. Specifically, the study hypothesizes that fear of radiation exerts a positive predictive effect on decisional conflict, with risk perception serving as a partial mediator in this relationship; additionally, it is anticipated that patient populations can be differentiated into several clinically meaningful subtypes based on combinatorial patterns of fear of medical imaging radiation. The theoretical contribution of this study lies in integrating the cognitive-affective dual-processing framework with risk perception theory to construct a psychological influence mechanism model within the medical imaging decision-making context, thereby filling a theoretical gap in this interdisciplinary domain. At the practical level, the research findings can provide evidence-based guidance for healthcare institutions to develop targeted patient education programs, risk communication strategies, and psychological support interventions, thereby advancing the realization of patient-centered imaging medicine service models.

## 2 Methods

### 2.1 Study design

This study employed a cross-sectional quantitative survey design that integrated both variable-centered and person-centered analytical approaches to investigate the mechanisms through which fear of medical imaging radiation influences patient decisional conflict, as well as the potential heterogeneity across patient populations. Research data were collected via electronic questionnaires. The questionnaire platform was equipped with quality control functions including logic skip patterns, mandatory response requirements, and duplicate submission restrictions to ensure standardization and completeness of data collection.

### 2.2 Participants

#### 2.2.1 Sample recruitment

This study employed convenience sampling to recruit participants from radiology departments, nuclear medicine departments, and imaging centers at two tertiary Grade-A general hospitals in Sichuan Province, China, between July and August 2025. Specifically, the research team established recruitment stations in the waiting areas of the aforementioned departments, where researchers explained the study purpose, content, and participation procedures to waiting patients. Patients willing to participate could access the electronic informed consent form by scanning a QR code. Upon fully understanding the research information and confirming voluntary participation, they proceeded to the formal questionnaire completion page. To enhance questionnaire recovery quality and completion rates, researchers provided participants who completed all valid questionnaires with gifts valued at 3 ¥as appreciation; this incentive measure received approval from the ethics committee.

#### 2.2.2 Inclusion and exclusion criteria

To further enhance research accuracy, this study established rigorous inclusion criteria for the study population. Specifically, inclusion criteria comprised: (1) adult patients aged 18 years or older; (2) patients currently scheduled to undergo medical imaging examinations; (3) possession of basic Chinese reading and writing abilities, enabling independent completion of electronic questionnaires or completion with researcher assistance; and (4) voluntary signing of informed consent and agreement to participate in this study.

Exclusion criteria comprised: (1) patients with severe mental disorders (such as schizophrenia or acute episodes of bipolar disorder) or cognitive impairment rendering them unable to comprehend questionnaire content; (2) personnel engaged in healthcare, nuclear energy, or radiation protection-related occupations; and (3) patients who explicitly expressed withdrawal during the questionnaire completion process.

It should also be noted that participant recruitment was conducted primarily in the waiting areas of radiology departments, nuclear medicine departments, and imaging centers. Therefore, the final sample predominantly consisted of patients who had already entered the imaging care pathway and were awaiting or preparing to undergo the prescribed examination. Accordingly, the present sample may not fully represent patients who had been advised to receive medical imaging but subsequently deferred or refused the examination before reaching the imaging department.

#### 2.2.3 Minimum sample size

For structural equation modeling, this study utilized G*Power software to conduct *a priori* statistical power analysis. Based on the theoretical model specifications of this study, we selected “Linear multiple regression: Fixed model, R² deviation from zero” from the F-test family as the test type to evaluate the overall explanatory power of latent variables on outcome variables in the model. Parameter settings were as follows: statistical power (1-β) was set at 0.80; significance level (α) was set at 0.05 (two-tailed test); effect size (f²) was set at 0.15 following Cohen’s standards, representing a medium effect size level based on meta-analytic results from previous studies on radiation fear and health decision-making. The number of predictor variables was set at 5 according to the core independent and mediating variables in this study’s structural model. G*Power calculations indicated a minimum required sample size of 138 cases. For latent profile analysis, Monte Carlo simulation studies by Nylund-Gibson and Choi (36) demonstrated that when the expected number of classes ranges from 3 to 5 and between-class separation is at a moderate level, the minimum sample size required to achieve stable and reliable class identification and parameter estimation is 300 to 500 cases.

Therefore, this study planned to recruit 400 to 500 participants to ensure that the final valid sample size would meet the statistical power requirements for all analyses while reserving adequate capacity for potential subgroup comparisons and sensitivity analyses.

#### 2.2.4 Data cleaning and final sample

A total of 500 questionnaires were distributed in this study. Given the questionnaire length, we first excluded data with abnormal completion times according to the following specific criteria: (1) questionnaire completion time shorter than 2 minutes was classified as careless responding; (2) response time exceeding 60 minutes was classified as attention interruption or non-continuous completion; both categories were excluded, totaling 13 participants. Second, we excluded questionnaires with patterned responding, including selection of the same option for all items, totaling 8 questionnaires. Additionally, 11 participants withdrew midway through completion, resulting in incomplete questionnaire responses that were excluded. Following this cleaning process, 468 valid questionnaires remained, yielding an effective response rate of 93.6%. Among these, 316 patients were male (67.50%) and 152 were female (32.50%). Participants’ educational levels were predominantly bachelor’s degree or above (N = 250, 53.40%). Patient ages ranged from 18 to 63 years, with a mean age of 28.93 years (SD = 6.957). Detailed demographic information is presented in **Table 1**.

**Table 1:** Summary of participant demographic information.

Variables	Items	Number (N)	Frequency
Gender	Male	316	67.50%
	Female	152	32.50%
Educational background	Primary school and below	27	5.80%
	top school to high school	191	40.80%
	Bachelor degree or above	250	53.40%
Place of residence	Cities	300	64.10%
	Rural area	168	35.90%
Marital status	Unmarried	269	57.50%
	Married	138	29.50%
	Divorced	57	12.20%
	Widowed	4	0.90%
Monthly income level	≤3000¥	53	11.30%
	3001-5000¥	188	40.20%
	5001-8000¥	185	39.50%
	8001 and above	42	9.00%
Medical imaging experience	No.	134	28.60%
	Yes.	334	71.40%
Types of medical imaging	Magnetic resonance Imaging	75	16.00%
	B-mode ultrasonography	76	16.20%
	CT	172	36.80%
	X-ray	110	23.50%
	Other	35	7.50%
Age	28.93 ± 6.957		

### 2.3 Ethical approval

This study strictly adhered to the relevant provisions of the Declaration of Helsinki. The research protocol was submitted to the Medical Ethics Committee of the researchers’ institution for review prior to formal data collection and received approval (No.: 2025ER447-1). All potential participants were required to read and sign an electronic informed consent form before formally completing the questionnaire. Research data collection was strictly confidential and anonymized.

### 2.4 Instruments tools

#### 2.4.1 Fear of medical imaging radiation scale

Items measuring fear of medical imaging radiation were derived from the Chinese version of the Fear of Medical Imaging Radiation Scale (FOMIRS) developed by Feng, She (11). This scale comprises 18 items distributed across two dimensions: the psychological dimension (FOMIR-P; 11 items) and the behavioral dimension (FOMIR-B; 7 items). The psychological dimension primarily measures individuals’ psychological perceptions, emotional distress, and cognitive biases regarding medical imaging radiation, while the behavioral dimension primarily measures behavioral tendencies and action patterns arising from fear. For example, “Do you agree that you believe standing outside the radiology department can still cause radiation harm?” This study used this scale to assess Chinese patients’ fear of medical radiation, employing a 5-point Likert scale ranging from 1 = strongly disagree to 5 = strongly agree. Higher scores indicate greater fear of medical imaging radiation. In this study, the Cronbach’s alpha coefficient for this scale was 0.936, indicating good internal consistency. Subsequently, confirmatory factor analysis demonstrated good model fit for this scale, with model fit indices presented in **Table 2**.

**Table 2:** Model fit indices and reliability of core study variables.

Variables	GFI	AGFI	CFI	RMSEA	χ²/df	TLI	Cronbach’s α
Fear of medical imaging radiation	0.894	0.866	0.930	0.067	3.066	0.921	0.936
Risk perception	0.932	0.914	0.953	0.049	2.124	0.946	0.921
Decisional conflict	0.931	0.910	0.946	0.056	2.440	0.937	0.913

#### 2.4.2 Risk perception scale

Items measuring risk perception were derived from the Tripartite Risk Perception Scale developed by Ferrer, Klein (37). This scale comprises 18 items distributed across three dimensions: the deliberative dimension (6 items), the affective dimension (6 items), and the experiential dimension (6 items). The scale was translated into Chinese by Zhou, Li (38) and validated for cultural adaptation and reliability among family members of patients with gastrointestinal cancer. Concurrently, this scale has been widely applied among Chinese patient populations, including older adults with sarcopenia (39) and patients with advanced cancer (40). Therefore, this study employed this scale to measure patients’ risk perception; for example, “Do you agree that you feel very worried about developing radiation-related diseases in the future?” This study used a 5-point Likert scale for scoring (1 = strongly disagree, 5 = strongly agree), with higher scores indicating greater risk perception. In this study, the Cronbach’s alpha coefficient for this scale was 0.921, indicating good internal consistency. Confirmatory factor analysis demonstrated good model fit for this scale, with model fit indices presented in **Table 2**.

#### 2.4.3 Decisional conflict scale

Items measuring decisional conflict were derived from the Mandarin version of the Decisional Conflict Scale (Chinese version) developed by Lu, Mu (41). This scale comprises 16 items distributed across five dimensions: informed subscale (3 items), values clarity subscale (3 items), support subscale (3 items), uncertainty subscale (3 items), and effective decision subscale (4 items). This scale has been widely applied among Chinese patient populations, including cancer survivors (42) and glaucoma patients (43), demonstrating good cultural adaptability and generalizability. This study employed this scale to measure patients’ decisional conflict when facing fear of medical imaging radiation; for example, “When you face medical imaging examinations, do you know what your options are?” A 5-point Likert scale was used for scoring (1 = strongly disagree, 5 = strongly agree), with total scores ranging from 16 to 80; higher scores indicate more severe decisional conflict. In this study, the Cronbach’s alpha coefficient for this scale was 0.913, indicating good internal consistency. Confirmatory factor analysis demonstrated good model fit for this scale, with model fit indices presented in **Table 2**.

### 2.5 Statistical analysis

This study employed SPSS 27.0, AMOS 30.0, and Mplus 8.3 software for statistical analyses. First, SPSS 27.0 software was used to conduct reliability testing for each measurement instrument, with Cronbach’s alpha coefficient employed to assess internal consistency of scales. AMOS 30.0 software was used to conduct confirmatory factor analysis to examine the structural validity of scales.

Second, Harman’s single-factor test was employed to examine common method bias. Concurrently, descriptive statistical analyses were conducted for demographic information and study variables. Categorical variables were expressed as frequencies (N) and percentages (%), while continuous variables were expressed as M ± SD with skewness and kurtosis calculated. Third, correlation analyses were used to examine the strength and direction of bivariate associations among continuous variables.

Regarding group comparisons, this study employed independent samples t-tests to compare mean differences in fear of radiation, risk perception, and decisional conflict across dichotomous demographic variables. For multi-categorical variables, one-way analysis of variance was used for between-group mean comparisons. Fifth, the PROCESS macro (Model 4) in SPSS 27.0 software was employed to verify the direct effect of fear of radiation on decisional conflict and the mediating effect of risk perception. Bootstrap resampling was set at 5,000 iterations, with bias-corrected confidence intervals selected as the confidence interval type. If confidence intervals did not contain zero, the mediation effect was considered to have reached statistical significance. Mediation effect size was reported using indirect effects and the proportion of mediation effect to total effect.

Sixth, Mplus 8.3 software was employed to conduct latent profile analysis to identify potential heterogeneous types of fear of medical imaging radiation among patients. In the analysis, models with 1 to 5 classes were fitted sequentially, with multiple indices used to comprehensively evaluate the optimal number of classes. Model fit and class selection evaluation indices with their interpretive criteria. After determining the optimal number of latent profile classes, one-way analysis of variance was used to conduct between-subgroup difference analyses.

## 3 Results

### 3.1 Common method bias

Given that all data in this study were derived from patient populations within the same province, the measurement context exhibited high similarity, and all assessments employed five-point Likert scales, the study potentially faced the threat of common method bias. To address this issue, multiple procedural control measures were implemented during the data collection phase. First, strict adherence to anonymization and confidentiality principles was maintained; the questionnaire’s first page explicitly informed participants that their responses would be kept completely confidential and would not involve any personally identifiable information, thereby reducing the influence of social desirability bias on responses. Second, measurement items from different constructs were randomly shuffled to avoid context effects or consistency motivation bias arising from consecutive presentation of measurement items.

To further examine common method bias, Harman’s single-factor test was employed for assessment. All items from all variables were subjected to exploratory factor analysis using principal component analysis to extract factors, and the unrotated factor solution structure was examined. Results revealed a total of six factors with eigenvalues greater than 1, among which the first common factor explained 34.130% of the variance, below the empirical threshold of 40%. This result indicates that the data in this study do not exhibit serious common method bias problems.

### 3.2 Descriptive statistics and correlation analysis

**Table 3** presents the descriptive statistical results and normality test indices for the three core variables in this study. Skewness values for core variables ranged from -0.032 to 0.030, and kurtosis values ranged from -0.093 to 0.035, meeting the data normality requirements for structural equation model analysis as proposed by Kline (44).

**Table 3:** Descriptive statistics and correlation analysis of core study variables.

Variables	M	SD	Kurtosis	Skewness	1	2	3
1. Fear of mediation imaging radiation	3.119	0.766	0.030	0.035	1		
2. Risk perception	3.189	0.716	0.014	-0.093	0.685**	2	
4. Decisional conflict	3.321	0.705	-0.032	0.015	0.628**	0.606**	3

M, mean; SD, standard deviation. **p < 0.01.

A significant positive correlation existed between fear of medical imaging radiation and risk perception (r = 0.685, p < 0.01), indicating that higher levels of patient radiation fear were associated with stronger subjective perceptions of radiation-related risks, with the strength of this association reaching a strong correlation level. Fear of medical imaging radiation and decisional conflict similarly exhibited a significant positive correlation (r = 0.628, p < 0.01), suggesting that patients with higher degrees of radiation fear were more likely to experience psychological conflict and uncertainty when facing imaging examination decisions. A significant positive correlation also existed between risk perception and decisional conflict (r = 0.606, p < 0.01), indicating that higher patient subjective assessments of radiation risk were associated with more pronounced conflict experiences during the decision-making process. Concurrently, correlation coefficients among variables did not exceed the critical value of 0.70, preliminarily ruling out the possibility of severe multicollinearity.

### 3.3 Between-group analysis

To explore differences in decisional conflict among patients with different demographic and clinical characteristics, this study employed independent samples t-tests and one-way analysis of variance for between-group comparisons; results are detailed in **Table 4**. Regarding gender, the difference in decisional conflict scores between male patients (M = 3.297, SD = 0.735) and female patients (M = 3.368, SD = 0.639) did not reach statistical significance (t = -1.02, p = 0.285), indicating that gender factors had limited influence on patient decisional conflict. Regarding educational level, significant differences in decisional conflict scores existed among different education groups (F = 9.324, p < 0.001, η² = 0.039). Specifically, patients with bachelor’s degrees or above had the highest decisional conflict scores (M = 3.445, SD = 0.744), followed by patients with junior high school to high school education (M = 3.200, SD = 0.655), while patients with elementary school education or below had the lowest scores (M = 3.028, SD = 0.387). This result suggests that patients with higher educational levels may face greater psychological conflict during decision-making, possibly due to having more access to health information channels and stronger risk awareness.

**Table 4:** Between-group analysis of demographic information and decisional conflict.

Variables	Items	M	SD	t/F	p	Cohen d/η2
Gender	Male	3.297	0.735	-1.02	0.285	0.705
	Female	3.368	0.639			
Educational background	Primary school and below	3.028	0.387	9.324	<0.001	0.039
	top school to high school	3.200	0.655			
	Bachelor degree or above	3.445	0.744			
Place of residence	Cities	3.414	0.703	3.898	<0.001	0.694
	Rural area	3.153	0.679			
Marital status	Unmarried	3.303	0.691	2.129	0.096	0.014
	Married	3.425	0.782			
	Divorced	3.161	0.548			
	Widowed	3.156	0.390			
Monthly income level	≤3000¥	3.037	0.725	3.855	0.010	0.024
	3001-5000¥	3.309	0.666			
	5001-8000¥	3.391	0.666			
	8001 and above	3.423	0.918			
Medical imaging experience	No.	3.147	0.597	-3.412	<0.001	0.697
	Yes.	3.390	0.733			
Types of medical imaging	Magnetic resonance Imaging	3.156	0.634	3.655	0.006	0.031
	B-mode ultrasonography	3.245	0.574			
	CT	3.323	0.680			
	X-ray	3.516	0.854			
	Other	3.209	0.590			

M, mean; SD, standard deviation; t, t-statistic for independent samples t-test; F, F-statistic for one-way analysis of variance (ANOVA); Cohen’s d, Cohen’s d effect size (reported for t-tests); η², eta-squared effect size (reported for ANOVA).

Urban patients (M = 3.414, SD = 0.703) had significantly higher decisional conflict scores than rural patients (M = 3.153, SD = 0.679) (t = 3.898, p < 0.001, Cohen’s d = 0.694), with this effect size reaching a medium level, indicating that urban-rural differences represent an important factor influencing patient decisional conflict. Differences in decisional conflict scores among different marital status groups did not reach statistical significance (F = 2.129, p = 0.096, η² = 0.014); although married patients’ decisional conflict scores (M = 3.425, SD = 0.782) were slightly higher than those of unmarried patients (M = 3.303, SD = 0.691), this difference may have resulted from sampling variability.

Regarding income level, significant differences in decisional conflict scores existed among different income groups (F = 3.855, p = 0.010, η² = 0.024). The group with monthly income of 8,001 Yuan or above had the highest decisional conflict scores (M = 3.423, SD = 0.918), while the group with monthly income of 3,000 Yuan or below had the lowest scores (M = 3.037, SD = 0.725). This result exhibits a consistent trend with findings regarding educational level, possibly reflecting that patients with higher socioeconomic status hold higher expectations for participation in medical decisions and greater information needs. Patients with prior imaging examination experience (M = 3.390, SD = 0.733) had significantly higher decisional conflict scores than patients without such experience (M = 3.147, SD = 0.597) (t = -3.412, p < 0.001, Cohen’s d = 0.697), with this effect size reaching a medium level. This finding suggests that prior radiation exposure experience may activate patients’ memories and concerns about radiation risks, thereby exacerbating psychological conflict in subsequent examination decisions.

Regarding medical imaging examination type, significant differences in decisional conflict scores existed among different examination type groups (F = 3.655, p = 0.006, η² = 0.031). Patients undergoing X-ray examinations had the highest decisional conflict scores (M = 3.516, SD = 0.854), while patients undergoing magnetic resonance imaging examinations had the lowest scores (M = 3.156, SD = 0.634). This difference may be related to the radiation characteristics of different examination types; as the most common radiation-based examination with the highest public awareness, X-ray may more readily trigger patients’ radiation-related concerns.

### 3.4 Mediating effect of risk perception

To examine the mediating role of risk perception in the relationship between fear of medical imaging radiation and decisional conflict, this study employed the PROCESS macro (Model 4) in SPSS 27.0 software to conduct mediation effect analysis. During the analysis, educational background, income level, residence location, medical imaging examination experience, and examination type were included as covariates for statistical control to eliminate confounding effects of these demographic and clinical characteristic variables on the core path relationships. Bootstrap resampling was set at 5,000 iterations, and bias-corrected percentile methods were used to construct 95% confidence intervals.

**Figure 1:**
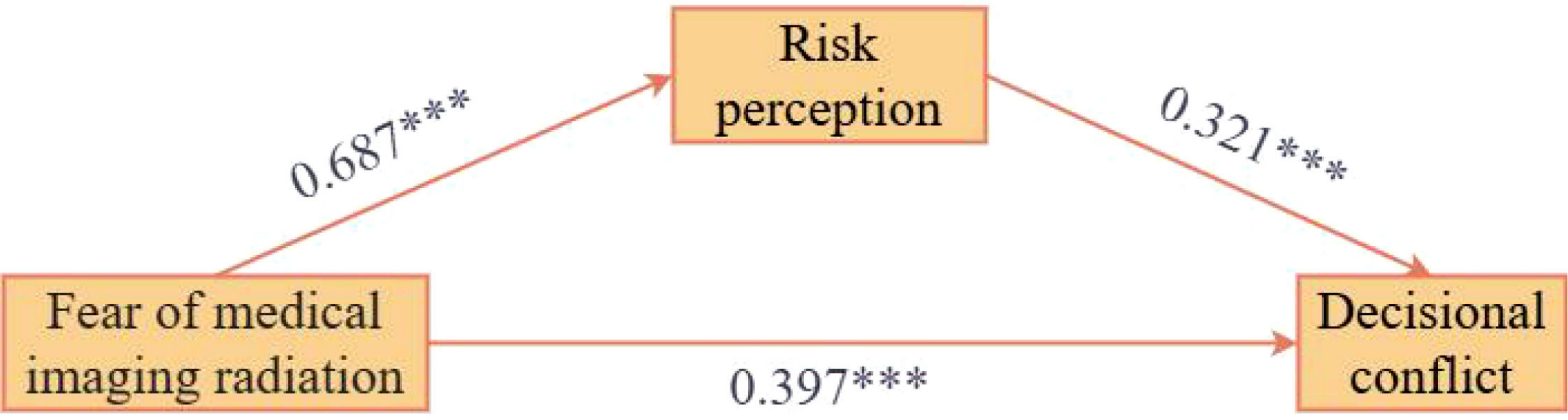
Mediation path coefficients of risk perception, ***p < 0.001.

**Table 5** and **Figure 1** presents the path coefficient results of the mediation effect test. After controlling for covariates, fear of medical imaging radiation was significantly positively associated with risk perception (β = 0.687, t = 20.229, p < 0.001, 95% CI = [0.579, 0.704]), indicating that for each one-unit increase in patients’ radiation fear level, their risk perception scores correspondingly increased by 0.687 units. The explanatory power of this path model reached 47.3% (R² = 0.473, F = 68.876), indicating that fear of medical imaging radiation and control variables could explain nearly half of the variance in risk perception.

**Table 5:** Regression coefficients of the risk perception mediation model.

Independent variable	Risk perception			Decisional conflict		
	β	t	95%CI	β	t	95%CI
Fear of medical imaging radiation	0.687	20.229***	[0.579,0.704]	0.397	8.767***	[0.284,0.448]
Risk perception				0.321	7.099	[0.229,0.404]
R^2^	0.473			0.504		
F	68.876			66.691		

β, standardized regression coefficient; t, t-statistic; CI, confidence interval; R², coefficient of determination; F, F-statistic for the overall regression model. ***p < 0.001.

In the model simultaneously incorporating fear of medical imaging radiation and risk perception to predict decisional conflict, fear of medical imaging radiation remained significantly associated with decisional conflict (β = 0.397, t = 8.767, p < 0.001, 95% CI = [0.284, 0.448]), while risk perception also demonstrated a significant positive predictive effect on decisional conflict (β = 0.321, t = 7.099, p < 0.001, 95% CI = [0.229, 0.404]). The explanatory power of this complete model was 50.4% (R² = 0.504, F = 66.691), representing an improvement over the model containing only direct effects, indicating that the inclusion of risk perception enhanced the model’s predictive capacity for decisional conflict.

**Table 6** presents the mediation effect decomposition and Bootstrap test results. The total effect of fear of medical imaging radiation on decisional conflict was 0.569 (SE = 0.032, 95% CI = [0.506, 0.631]). Upon decomposition of the total effect, the direct effect was 0.366 (SE = 0.042, 95% CI = [0.284, 0.448]), accounting for 64.32% of the total effect; the indirect effect transmitted through risk perception was 0.203 (SE = 0.065, 95% CI = [0.097, 0.349]), accounting for 35.68% of the total effect. Since the 95% Bootstrap confidence interval for the indirect effect did not contain zero, it can be confirmed that risk perception plays a significant partial mediating role between fear of medical imaging radiation and decisional conflict. In summary, the mediation effect test results support the core hypothesis of this study: fear of medical imaging radiation not only directly increases patients’ decisional conflict but also indirectly exacerbates the decisional conflict experience by elevating patients’ risk perception levels. Risk perception exerts a partial mediating effect in the relationship between the two, with the proportion of mediation effect to total effect being approximately one-third.

**Table 6:** Effect decomposition of the mediation model.

Type of effect	β	SE	LLCI	ULCI	Proportion of effect
Total effect	0.569	0.032	0.506	0.631	100%
Direct effect	0.366	0.042	0.284	0.448	64.32%
Indirect effect	0.203	0.065	0.097	0.349	35.68%

LLCI, lower limit of 95% confidence interval; ULCI, upper limit of 95% confidence interval.

### 3.5 Latent profile analysis of fear of medical imaging radiation

To identify potential heterogeneous types in fear of medical imaging radiation characteristics among patient populations, this study employed Mplus 8.3 software to conduct latent profile analysis. Using scores on the psychological dimension and behavioral dimension of the Fear of Medical Imaging Radiation Scale as profile indicator variables, latent profile models with 1 to 5 classes were fitted sequentially; fit indices for each model are detailed in **Table 7**.

**Table 7:** Fit indices for latent profile models with different numbers of classes.

Profile	AIC	BIC	aBIC	Entropy	LMR (p)	BLRT (p)	Smallest proportion per class				
							1	2	3	4	5
1	25676.805	25826.150	25711.893	-	-	-	-				
2	23425.216	23653.382	23478.823	0.917	<0.001	<0.001	64.2%	35.8%			
3	22347.676	22654.663	22419.802	0.939	<0.001	<0.001	19.4%	60.1%	20.5%		
4	22068.289	22454.096	22158.934	0.877	0.456	<0.001	11.7%	30.4%	40.9%	16.9%	
5	21964.748	22429.376	22073.912	0.865	0.185	<0.001	10.3%	14.9%	19.5%	39.7%	15.5%

As the number of classes increased, AIC, BIC, and aBIC values all exhibited declining trends, indicating progressive improvement in model fit. However, the decline from the 2-class model to the 3-class model was relatively pronounced, while declines from the 3-class model to the 4-class model and from the 4-class model to the 5-class model tended to level off, suggesting that the 3-class model may have reached an optimal fit level. Regarding likelihood ratio test indices, both LMR-LRT and BLRT tests for the 2-class model reached significance (p < 0.001), indicating that the 2-class model was significantly superior to the 1-class model; LMR-LRT and BLRT tests for the 3-class model were similarly significant (p < 0.001), indicating that the 3-class model was significantly superior to the 2-class model. However, the LMR-LRT test for the 4-class model did not reach significance (p = 0.456); although the BLRT test remained significant (p < 0.001), the non-significant LMR-LRT result suggested that adding a fourth class did not yield substantive model improvement. The LMR-LRT test for the 5-class model did not reach significance (p = 0.185), further supporting the superiority of the 3-class model.

From the classification accuracy index perspective, the 3-class model had the highest entropy value (Entropy = 0.939), meeting the criterion for excellent classification accuracy and indicating that this model could clearly differentiate patients across different classes. In comparison, entropy values for the 4-class model (Entropy = 0.877) and 5-class model (Entropy = 0.865), while still at good levels, showed decreases relative to the 3-class model. The smallest class in the 3-class model comprised 19.4% of the sample, far exceeding the minimum threshold requirement of 5%, ensuring that each class had sufficient sample size to support subsequent statistical analyses.

Therefore, based on multiple evaluation indices and theoretical interpretability of classes, this study determined the 3-class model as the optimal latent profile solution.

**Figure 2:**
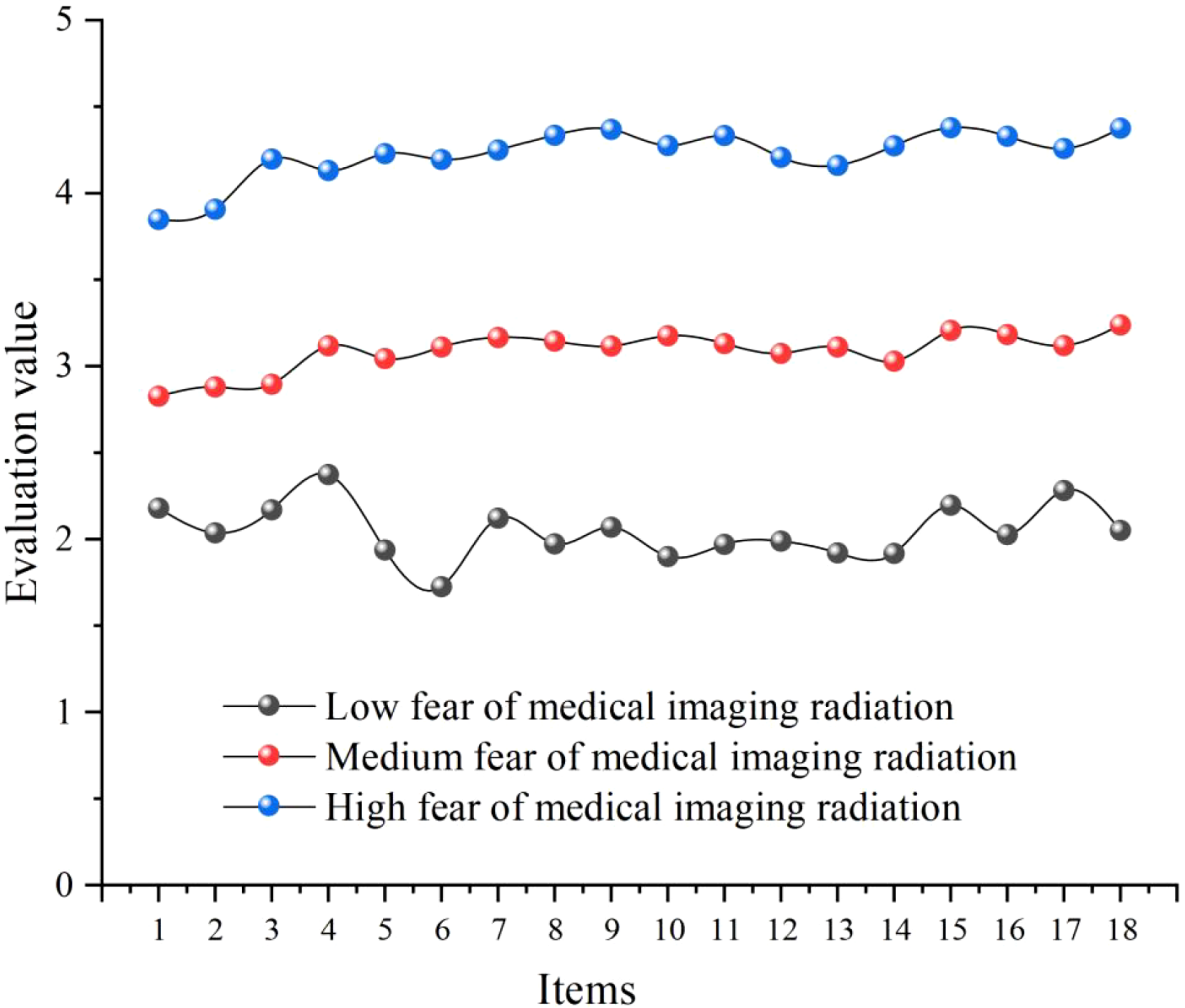
Profile plot of latent subgroups of fear of medical imaging radiation.

**Figure 2** presents the scoring patterns across the psychological and behavioral dimensions of fear of medical imaging radiation for each class in the 3-class latent profile model. The first latent subgroup was the low radiation fear group (n = 91, 19.4%). Patients in this class exhibited relatively low score levels on both the psychological and behavioral dimensions of radiation fear, representing a patient group that holds a relatively calm attitude toward medical imaging radiation. These patients may possess better health literacy or prior positive medical experiences, enabling them to rationally view radiation exposure from diagnostic imaging examinations with minimal tendencies toward excessive worry or avoidance behaviors.

The second latent subgroup was the moderate radiation fear group (n = 281, 60.1%). This class represented the largest proportion of patients in this sample, with scores at moderate levels on both dimensions of radiation fear. These patients harbor a certain degree of concern about medical imaging radiation but have not yet developed significant psychological distress or behavioral impairment. This class represents the typical cognitive and emotional response pattern toward radiation risk among the general patient population.

The third latent subgroup was the high radiation fear group (n = 96, 20.5%). Patients in this class exhibited relatively high score levels on both the psychological and behavioral dimensions of radiation fear, representing a high-risk group that holds intense fear regarding medical imaging radiation. These patients may exhibit excessive worry about radiation hazards, catastrophizing thinking tendencies, and obvious examination avoidance behaviors, constituting a target population requiring focused clinical intervention attention.

### 3.6 Differential analysis of latent subgroups

**Figure 3:**
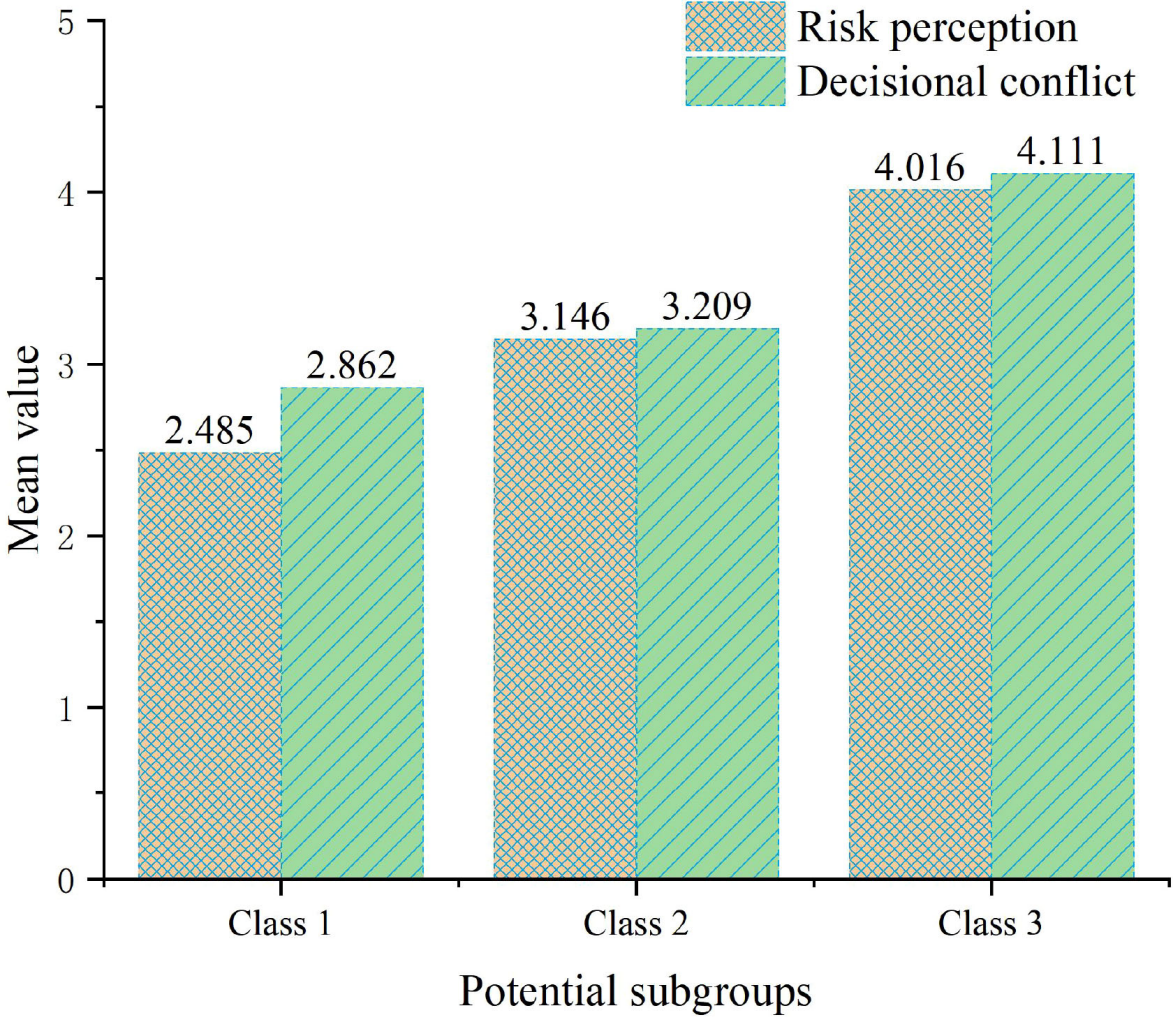
One-way ANOVA of potential subgroups and risk perception and decision conflict. Class 1: low radiation fear group; Class 2: Medium radiation fear group; Class 3: High radiation fear group.

To further explore the differential characteristics of patients with different radiation fear types regarding risk perception and decisional conflict, this study employed one-way analysis of variance to compare score differences across the three latent subgroups on these two outcome variables; results are detailed in **Table 8** and **Figure 3**.

**Table 8:** One-way analysis of variance for latent subgroups, risk perception, and decisional conflict.

Outcome variable	Index	Latent subgroups		
		Class 1	Class 2	Class 3
Risk perception	M	2.485	3.146	4.016
	SD	0.738	0.404	0.609
	p	<0.001		
	F	196.661		
	η2	0.348		
Decisional Conflict	M	2.863	3.209	4.111
	SD	0.834	0.437	0.608
	p	<0.001		
	F	124.298		
	η2	0.458		

Class 1, low radiation fear group; Class 2, Medium radiation fear group; Class 3, High radiation fear group.

Regarding risk perception, significant differences existed among the three latent subgroups (F = 196.661, p < 0.001, η² = 0.348). The low radiation fear group had the lowest risk perception scores (M = 2.485, SD = 0.738), followed by the moderate radiation fear group (M = 3.146, SD = 0.404), while the high radiation fear group had the highest scores (M = 4.016, SD = 0.609). The effect size η² = 0.348 indicates that latent class membership could explain 34.8% of the variance in risk perception, reaching a large effect size level. *Post-hoc* multiple comparison results showed that pairwise differences among all three classes reached statistical significance (p < 0.001), indicating that risk perception levels across different radiation fear type patients exhibited clear gradient distribution characteristics.

Regarding decisional conflict, significant differences similarly existed among the three latent subgroups (F = 124.298, p < 0.001, η² = 0.458). The low radiation fear group had the lowest decisional conflict scores (M = 2.863, SD = 0.834), followed by the moderate radiation fear group (M = 3.209, SD = 0.437), while the high radiation fear group had the highest scores (M = 4.111, SD = 0.608). The effect size η² = 0.458 indicates that latent class membership could explain 45.8% of the variance in decisional conflict, similarly reaching a large effect size level, with explanatory power even exceeding that for risk perception. *Post-hoc* multiple comparison results similarly showed that pairwise differences among all three classes reached statistical significance (p < 0.001).

## 4 Discussion

### 4.1 Variable-centered analysis

This study found that fear of medical imaging radiation was significantly positively associated with patient decisional conflict. After controlling for covariates including educational background, income level, residence location, and medical imaging examination experience, the total effect of radiation fear on decisional conflict reached 0.569. This result is consistent with previous research findings regarding the influence of health anxiety on medical decision-making. The risk-as-feelings hypothesis proposed by Slovic (45) provides a theoretical foundation for understanding this association; this hypothesis posits that individuals’ judgments about risk are often strongly influenced by immediate emotional reactions rather than solely relying on rational probability calculations. Due to characteristics such as invisibility, uncontrollability, and potential association with cancer, medical imaging radiation readily triggers fear emotions in patients, subsequently interfering with information integration and value trade-offs during their decision-making process.

More importantly, this study confirmed that risk perception plays a significant partial mediating role in the relationship between radiation fear and decisional conflict. This finding extends our understanding of the internal mechanisms through which radiation fear influences the decision-making process. According to the Health Belief Model and Protection Motivation Theory, fear emotions activate individuals’ threat appraisal systems, enhancing their subjective perceptions of disease susceptibility and severity, which subsequently influence behavioral decisions (46). In the context of medical imaging examinations, radiation fear may first activate patients’ cognitive schemas regarding negative consequences such as radiation-induced cancer and genetic damage, amplifying their subjective assessment of examination risks and generating greater psychological conflict when patients weigh examination benefits against potential risks. The revelation of this mediating pathway provides a clear intervention target for clinical practice, suggesting that correcting patients’ cognitive biases regarding radiation risk may effectively alleviate their decisional dilemmas.

Notably, the direct effect of radiation fear on decisional conflict remained significant and accounted for 64.32% of the total effect, indicating that psychological factors other than risk perception may also help explain the association between radiation fear and decisional conflict. One possible explanation is that fear emotions themselves possess direct cognitive interference functions (47). According to Attentional Control Theory (48), anxiety and fear emotions consume individuals’ limited cognitive resources, reducing their ability to inhibit threat-related irrelevant information, leading to decreased information processing efficiency during decision-making. Furthermore, individuals in fear states may be more inclined to adopt heuristic rather than analytical information processing modes (49); while this processing approach enables rapid judgments, it is also more susceptible to cognitive biases, thereby exacerbating feelings of decisional uncertainty. Future research may further explore the roles of variables such as cognitive load and information processing modes in the relationship between radiation fear and decisional conflict, to more comprehensively delineate the psychological mechanism landscape of this influence process.

Patients with higher educational levels and those residing in urban areas exhibited higher levels of decisional conflict; this seemingly paradoxical result actually reflects the double-edged sword effect of health information literacy. On one hand, higher educational levels and urban living environments provide patients with easier access to diverse information sources regarding radiation hazards, including popular science articles, social media discussions, and peer experience sharing. On the other hand, such information often varies considerably in quality and may even be contradictory, potentially increasing patients’ cognitive confusion and decisional uncertainty. The finding that patients with prior imaging examination experience exhibited higher levels of decisional conflict similarly warrants attention; this may stem from prior examination experiences activating patients’ cumulative concerns about radiation exposure, or from unpleasant prior examination experiences forming negative emotional memories that are automatically evoked when facing subsequent examination decisions. Differences in decisional conflict among different examination types suggest that significant disparities exist in public awareness of radiation characteristics across various medical imaging examinations; as the most widely known radiation-based examination, X-ray may carry greater public concern.

### 4.2 Person-centered analysis

This study employed latent profile analysis to identify three patient subtypes with distinct radiation fear characteristics. This finding reveals significant heterogeneity within patient populations from a person-centered perspective, providing an important supplement to traditional variable-centered analysis. The low radiation fear group comprised 19.4% of the sample; patients in this class exhibited low fear levels on both psychological and behavioral dimensions. This group may represent patients with better psychological resilience or higher health literacy who can rationally recognize the risk-benefit ratio of diagnostic medical imaging examinations and are less troubled by irrational fears. From a clinical practice perspective, these patients can typically complete the informed consent process relatively smoothly, and clinician-patient communication can focus on explaining examination procedures and interpreting results without requiring excessive resources for fear alleviation.

The moderate radiation fear group was the largest in this sample, comprising 60.1%. Patients in this class harbor a certain degree of concern about medical imaging radiation but have not yet developed significant psychological impairment or behavioral avoidance. This distribution characteristic aligns with the general public’s cognitive status regarding radiation risk, reflecting normal psychological responses of the general population when facing uncertain health threats. For this mainstream group, standardized patient education and risk communication strategies may be sufficient to meet their information needs, although vigilance remains necessary regarding the possibility of some patients transitioning toward the high fear type under specific circumstances. The high radiation fear group comprised 20.5% of the sample, meaning approximately one-fifth of patients belonged to this type. Patients in this class exhibited high scores on both dimensions of radiation fear; more importantly, their risk perception and decisional conflict levels were also significantly higher than the other two groups. Effect size analysis showed that latent class membership could explain 45.8% of the variance in decisional conflict. Patients in the high radiation fear group may exhibit catastrophizing thinking tendencies, subjectively amplifying low-probability radiation harm events into high-probability threats, and may simultaneously exhibit obvious examination avoidance motivation. From a clinical perspective, this group represents the target population most requiring attention and intervention, as excessive radiation fear may lead them to delay or refuse necessary imaging examinations, subsequently affecting timely disease diagnosis and treatment.

The three subgroups exhibited clear gradient distribution characteristics in risk perception and decisional conflict, with scores increasing progressively from low to moderate to high fear groups, and pairwise differences all reaching statistical significance. This finding further validates, from a person-centered perspective, the radiation fear–risk perception–decisional conflict chain revealed by variable-centered analysis. Notably, the larger difference observed between the high and moderate fear groups may indicate that the association between radiation fear and decisional conflict varies across levels of fear. Nevertheless, this interpretation remains tentative because nonlinear relationships were not directly tested in the present study.

### 4.3 Clinical and practical implications

The findings of this study offer multi-level implications for optimizing patient management and clinician-patient communication practices in medical imaging examinations. At the level of patient screening and risk identification, the research results support the necessity of introducing rapid radiation fear screening tools in imaging department waiting areas. Given that approximately one-fifth of patients belong to the high radiation fear type and this group exhibits significantly elevated decisional conflict levels, early identification of these high-risk patients helps healthcare personnel adjust communication strategies and time allocation in a timely manner. Screening tools may adopt simplified versions of radiation fear scales or structured clinical interview questions, completing preliminary assessment during patient waiting periods; patients whose screening results indicate high fear levels may be scheduled for dedicated clinician-patient communication time or psychological support resources. For patients with different radiation fear subtypes, clinical practice should adopt differentiated communication and support strategies: for patients in the low radiation fear group, standardized informed consent procedures typically meet their needs; for patients in the moderate radiation fear group, adding brief radiation safety explanations and question-answering sessions to standard procedures may help further reduce their concern levels; for patients in the high radiation fear group, greater communication time and emotional support resources are needed, and involvement of mental health professionals may be considered when necessary. Communication strategies should avoid purely didactic information delivery, instead employing empathic listening and motivational interviewing techniques, first acknowledging the legitimacy of patients’ fear emotions, then guiding them to rationally examine biased components in their risk perceptions.

The mediating effect of risk perception revealed in this study suggests that correcting patients’ cognitive biases regarding radiation risk represents an effective pathway for alleviating their decisional conflict. Various strategies may be employed in clinical practice to achieve this goal: providing dose comparison information can help patients establish reference frameworks for radiation risk, such as comparing the radiation dose of a single chest X-ray examination with daily background radiation or airplane travel radiation, making abstract dose figures more concrete; emphasizing the diagnostic value of examinations and potential risks of delayed examination helps patients conduct more balanced risk-benefit trade-offs, avoiding excessive focus on radiation risk while neglecting the urgency of disease diagnosis; employing visual information presentation methods such as charts and infographics may more effectively convey risk information than pure text explanations, especially for patient populations with lower health literacy levels. The findings of this study similarly provide empirical evidence for designing patient decision aids for medical imaging examinations. Effective decision aids should integrate clear explanations of examination indications and diagnostic value, objective information about radiation doses and potential risks, comparative explanations of alternative examination options, and guiding questions to help patients clarify personal values and preferences. Such tools may adopt various forms including paper booklets, electronic applications, or interactive web pages, provided for use during patient waiting periods or examination scheduling phases.

The results of this study suggest the necessity of strengthening risk communication competency training for healthcare personnel in radiology and related departments. Training content should encompass the psychological mechanisms and manifestations of radiation fear, common bias types in patient risk perception, effective risk communication techniques with example scripts, and strategies and resources for managing high-fear patients. Emphasis should also be placed on healthcare personnel’s own accurate understanding of radiation risk, avoiding inadvertently exacerbating patient concerns through inappropriate expressions. At the healthcare institution level, research findings support establishing systematic patient psychological support systems in imaging departments. Such systems may include optimized waiting environment design to reduce patient anxiety arousal levels, standardized development and continuous updating of patient education materials, identification and referral procedures for high-fear patients, and collaborative mechanisms with psychology departments or social work departments. For patients who persistently refuse necessary examinations due to severe radiation fear, multidisciplinary consultation mechanisms should be established to comprehensively assess their psychological status and medical needs, formulating individualized diagnostic and treatment plans, ultimately serving the goal of improving patient medical decision quality and health outcomes.

### 4.4 Limitations and future research directions

Although this study achieved several meaningful findings, the following limitations remain. First, this study employed a cross-sectional survey design; while this effectively tests association patterns among variables, it cannot establish causal inference. Future research should adopt longitudinal tracking designs, collecting data at multiple time points before and after patients undergo imaging examinations, to clarify temporal relationships and dynamic change trajectories among variables. Additionally, experimental or quasi-experimental designs may be used to test the effectiveness of targeted interventions (such as risk communication strategies and decision aids) in alleviating patient radiation fear and decisional conflict.

Second, this study’s sample was derived from two tertiary Grade A general hospitals in Sichuan Province, China, presenting certain limitations in geographic representativeness and institutional type diversity. Patient populations at tertiary Grade A hospitals may differ from patients at primary healthcare institutions in aspects such as disease severity, healthcare resource accessibility, and health literacy levels, which may affect the external generalizability of research conclusions. Future research should expand the geographic coverage and healthcare institution tier distribution of samples to verify the applicability of this study’s findings across different contexts. Cross-cultural comparative studies are also worth conducting to explore whether cultural differences exist in the psychological mechanisms of radiation fear.

Meanwhile, the limitation concerns the heterogeneity of imaging modalities included in the sample. Although the focal construct of this study was fear of medical imaging radiation, the sample included patients scheduled for both ionizing (e.g., X-ray and CT) and non-ionizing (e.g., MRI and ultrasonography) examinations, because recruitment was conducted in broader imaging-care settings. This approach reflects the clinical reality that some patients may hold generalized or inaccurate beliefs about “radiation” across imaging modalities; however, it also introduces conceptual heterogeneity. Therefore, the findings should be interpreted as most directly relevant to radiation-related fear in imaging contexts, rather than as reflecting actual exposure characteristics of each modality per se. Future studies should distinguish more explicitly between ionizing and non-ionizing imaging groups and examine whether the observed associations differ across these contexts.

Third, all variables in this study were measured through self-report questionnaires, potentially subject to influences from common method bias, social desirability bias, and recall bias. Although Harman’s single-factor test results indicated that common method bias problems were not severe, its influence cannot be completely ruled out. Future research may consider integrating multiple measurement methods, such as employing physiological indicators (e.g., galvanic skin response, heart rate variability) to measure somatic response components of fear, using behavioral observation to record patients’ actual examination completion status, or employing implicit association tests to assess patients’ implicit attitudes toward radiation.

Fourth, this study only examined the role of risk perception as a single mediating variable, while the psychological mechanisms through which radiation fear influences decisional conflict may involve multiple parallel or sequential mediating pathways. For example, physician-patient trust may influence patients’ acceptance of physicians’ radiation safety assurances, self-efficacy may influence patients’ ability to cope with fear emotions, and information-seeking behavior may influence patients’ knowledge structures and degrees of cognitive bias. Future research may construct more complex multiple mediation or moderated mediation models to more comprehensively delineate the psychological mechanism network of this influence process. Exploration of moderating variables similarly warrants attention; for example, patient personality traits (such as neuroticism and openness), physician communication styles, and healthcare environment characteristics may moderate the strength of radiation fear’s influence on decisional conflict.

An additional limitation concerns the sampling frame of the present study. Participants were recruited mainly from imaging-related waiting areas, which means that the sample largely represented patients who ultimately proceeded to the examination process. Patients who were prescribed or advised to undergo medical imaging but decided to defer or refuse the examination were not systematically captured in the current design. This issue is important because the level and structure of decisional conflict may differ between imaging accepters and imaging non-accepters. On the one hand, patients who opt out of imaging may experience stronger radiation fear, heightened risk perception, and greater decisional conflict, which could make them particularly vulnerable to delayed diagnosis and treatment. On the other hand, some patients who refuse imaging may have already formed a clear and stable preference against the examination, in which case their decisional conflict may not necessarily be higher, even though their behavioral choice differs. Therefore, the present findings should be interpreted as being most applicable to patients who had already entered the imaging pathway, rather than to all patients facing imaging-related decisions. Future studies should purposively recruit and compare at least two groups—patients who complete recommended imaging and patients who defer or refuse it—to determine whether the magnitude, configuration, and psychological mechanisms of decisional conflict differ across these clinically important groups.

Finally, the latent profile analysis in this study was based only on two dimensions of radiation fear as indicator variables, without incorporating risk perception and decisional conflict as profile indicators. Future research may consider employing latent transition analysis to track patients’ dynamic transition patterns among different radiation fear types, or employing multi-indicator latent profile analysis to simultaneously examine combination pattern types of radiation fear, risk perception, and decisional conflict, to obtain richer understanding of patient heterogeneity.

## 5 Conclusion

Through a cross-sectional survey of 468 Chinese patients, this study employed a cross-sectional quantitative design combining regression-based mediation analysis and latent profile analysis to systematically investigate the mechanisms through which fear of medical imaging radiation influences patient decisional conflict and the heterogeneity characteristics of patient populations. The study found that fear of medical imaging radiation has a significant positive predictive effect on patient decisional conflict; patients with higher radiation fear levels experience more intense psychological conflict when facing imaging examination decisions. More importantly, risk perception plays a significant partial mediating role in the relationship between fear of medical imaging radiation and decisional conflict, with the mediating effect accounting for 35.68% of the total effect; radiation fear indirectly exacerbates patients’ decisional conflict experience by elevating their subjective risk assessment of radiation hazards. Latent profile analysis further identified three clinically meaningful radiation fear subtypes: the low radiation fear group (19.4%), the moderate radiation fear group (60.1%), and the high radiation fear group (20.5%). The three subgroups exhibited significant gradient differences in risk perception and decisional conflict levels, with the decisional conflict level of patients in the high radiation fear group being particularly pronounced. The findings of this study reveal the mechanistic role of risk perception as a key mediating variable and the significant heterogeneity characteristics within patient populations, providing evidence-based foundations for healthcare institutions to develop targeted patient education programs, risk communication strategies, and psychological support interventions.

## Data Availability

The raw data supporting the conclusions of this article will be made available by the authors, without undue reservation.
